# Trends and inequalities in short-term acute myocardial infarction case fatality in Scotland, 1988-2004

**DOI:** 10.1186/1478-7954-8-33

**Published:** 2010-12-06

**Authors:** Carolyn A Davies, Alastair H Leyland

**Affiliations:** 1 MRC/CSO Social and Public Health Sciences Unit, 4 Lilybank Gardens, Glasgow, UK, G12 8RZ

## Abstract

**Background:**

There have been substantial declines in ischemic heart disease in Scotland, partly due to decreases in acute myocardial infarction (AMI) incidence and case fatality (CF). Despite this, Scotland's IHD mortality rates are among the worst in Europe. We examine trends in socioeconomic inequalities in short-term CF after a first AMI event and their associations with age, sex, and geography.

**Methods:**

We used linked hospital discharge and death records covering the Scottish population (5.1 million). Between 1988 and 2004, 178,781 of 372,349 patients with a first AMI died on the day of the event (Day0 CF) and 34,198 died within 28 days after surviving the day of their AMI (Day1-27 CF).

**Results:**

Age-standardized Day0 CF at 30+ years decreased from 51% in 1988-90 to 41% in 2003-04. Day1-27 CF decreased from 29% to 18% over that period. Socioeconomic inequalities in Day0 CF existed for both sexes and persisted over time. The odds of case fatality for men aged 30-59 living in the most deprived areas in 2000-04 were 1.7 (95%CI: 1.3-2.2) times as high as in the least deprived areas and 1.9 (1.1-3.2) times as high for women. There was little evidence of socioeconomic inequality in Day1-27 CF in men or women. After adjustment for socioeconomic deprivation, significant geographic variation still remained for both CF definitions.

**Conclusions:**

A high proportion of AMI incidents in Scotland result in death on the day of the first event; many of these are sudden cardiac deaths. Short-term CF has improved, perhaps reflecting treatment advances and reductions in first AMI severity. However, persistent socioeconomic and geographic inequalities suggest these improvements are not uniform across all population groups, emphasizing the need for population-wide primary prevention.

## Introduction

Declining ischemic heart disease (IHD) mortality in the world's developed regions can be partly explained by decreasing incidence of the disease, suggesting effective primary prevention measures, and partly by reduced case fatality rates, reflecting improved primary and secondary care [1]. Downward trends in IHD mortality have been seen in Scotland [2], but to a lesser extent than in other Western European countries, resulting in Scotland having one of the worst IHD mortality rates in the region [3]. Incidence [2] and case fatality [4-7] from the disease have also been declining rapidly in Scotland, but its fatal and nonfatal event rates remain high when compared internationally [8]. The overall picture for survival is not so bleak, with 28-day case fatality following an acute myocardial infarction (AMI) in Scotland shown to be the same as or lower than the average across all populations monitored by the World Health Organization MONICA Project [8]. However, for Scotland to achieve its health potential, it is important that the downward trends in AMI case fatality are experienced by all sectors of society.

Despite large reductions in rates, there remain strong regional differences [9,10] and socioeconomic inequalities [10,11] in IHD mortality in Scotland. This partly reflects increasing socioeconomic and geographic variations in AMI incidence [2] as well as similar patterning of AMI case fatality. The majority of Scottish studies exploring the effect of deprivation on AMI case fatality have shown that socioeconomic inequalities exist [5-7,12-14]. Such inequalities also contribute to the gradient in IHD mortality. Studies outside Scotland have provided conflicting evidence, with some showing evidence of a socioeconomic gradient in AMI case fatality whose strength is dependent on whether the focus is on in-hospital [15-17] or out-of-hospital [15,18] AMI events. Some work, however, has suggested that the associations between short-term mortality and area deprivation or education are weak and inconsistent [19].

Further, in Scotland, there is conflicting evidence of sex differences in the socioeconomic inequalities in short-term AMI case fatality. For example, in terms of hospital admissions, inequalities in one-month case fatality have been shown to exist in men but not women [7], but also to be stronger in women than in men [13]. The most up-to-date study comparing socioeconomic inequalities between males and females examined AMI events up to 1995. The extent of socioeconomic differences and the Scottish government's commitment to tackling inequalities in health emphasize the importance of exploring whether similar declines in case fatality rates over recent years have been experienced by all population groups.

This study was designed to explore socioeconomic inequalities in short-term AMI case fatality and, in particular, to examine any temporal changes and associations with age, sex, and geography. Specifically, we will address three hypotheses. We hypothesize that a socioeconomic inequality pattern will exist for one-day case fatality, and that, similar to AMI incidence inequalities [2], this pattern will persist over time and will be steeper at younger ages and steeper for women than for men. For days 1-27, we hypothesize that no socioeconomic inequalities will exist. Finally, we hypothesize that any geographical variations in case fatality will be explained by the patterns of socioeconomic deprivation in Scotland.

Population-based studies in AMI case fatality are fairly uncommon, but Scotland has the advantage of having the only morbidity record database in the United Kingdom that routinely links information on all hospital admissions with all mortality data for a geographically defined population, the 5.1 million people in Scotland [20]. The aim of this work is to examine the trends and inequalities in short-term case fatality after a first AMI event in Scotland between 1988 and 2004. Our data contain accurate information from 1981 to 2004 on 1,035,692 individual IHD events, of which 457,363 resulted in deaths.

## Methods

### Data source

The data were obtained from the Scottish system of hospital discharge records. The Information and Services Division of the National Health Service in Scotland routinely links these records to mortality data provided by the General Register Office for Scotland [21]. Incidence was defined as a first-time attack within a seven-year period, with AMI (ICD9: 410, ICD10: I21-I22) as primary or secondary diagnosis at discharge or underlying or contributory cause of death or other IHD (ICD9:410-414; ICD10: I20-I25) as underlying cause of death [22]. Linkage enabled us to identify 375,848 individuals aged 30+ years who had an incident event between 1988 and 2004. Short-term case fatality was defined as the proportion of AMI incident events in which the patient died from any cause within a 28-day period. More specifically, Day0 means death on the day of the event, with a denominator of all incident events, and Day1-27 means death within 28 days, with a denominator of all who survived the day of the event. Each patient record provides information on age, sex, postcode, and Health Board (HB) of residence, date of admission, discharge, and death, if it occurred. Postcode sectors (mean population 5402, range 53-20,512) were used to allocate patients into seven deprivation categories (DEPCAT) using Carstairs socioeconomic deprivation scores [23]. Throughout the period, 3,499 patient records (1%) had missing postcode information and were excluded from our analysis.

### Statistical analysis

Case fatality is defined as the proportion of events ending fatally within a defined period from the onset of an attack [24] and is therefore modeled using logistic regression. We analyzed the data using multilevel modeling [25] in MLwiN [26] to take account of the hierarchical data structure. Individuals are nested within postcode sectors within HBs; the rates for postcode sectors within the same HB are likely to be correlated, as is case fatality for individuals within a given postcode sector. Adjustment was made for age, sex, year of first AMI event, DEPCAT (1-least; 7-most), and significant interactions between these. Odds ratios were used to assess the effect of these factors on AMI case fatality rates. Directly standardized rates are also presented within these strata.

Geographic inequalities were assessed using the intraclass correlation coefficient [27], which partitions the total variation in case fatality to that attributable to each of postcode sector (n = 1,010) and HB (n = 15) levels. A larger variance at a given level indicates greater geographical inequality. Larsen and Merlo [28] discuss the disadvantages of using the intraclass correlation when examining binary responses and propose the use of the median odds ratio (MOR) as an alternative. These are also presented. The MOR quantifies the variation between areas by comparing two persons with identical characteristics from two different, randomly chosen areas. It is the median of the odds ratios obtained between the person from the area with higher propensity of case fatality and the person from the area with lower propensity.

## Results

### Baseline characteristics of incidents and case fatalities of AMI

The number of people who experienced their first AMI (372,349) between 1988 and 2004 is shown in Table 1 broken down by sex, age, deprivation, and year. Alongside these figures are numbers and percentages of those who died on the day of their AMI and those who died within 28 days. Between 1988 and 2004, 178,781 patients with a first AMI died on the day of their event (crude Day0 case fatality 48%), and of the 192,568 patients who survived the day of their first AMI, 34,198 died within 28 days (crude Day1-27 case fatality 18%). Age-standardized Day0 case fatality decreased from 51% in 1988-90 to 41% in 2003-04, and Day1-27 decreased from 29% to 18% over the same time period. For each case fatality definition, standardized rates are shown, and univariable models were fitted to explore the relationships with sex, age, DEPCAT, and year. Sex significantly affects each definition. Women have significantly higher short-term case fatality rates than men, and this sex difference is strongest in Day1-27 case fatality. As expected, age is strongly related to case fatality. The chances of short-term survival are reduced significantly as age increases. The effect of deprivation was unclear from the unadjusted results. There was a clear downward time trend, with the odds of case fatality decreasing significantly as year increases.

**Table 1:** Baseline characteristics of AMI incidence, counts (and %) of case fatality (CF), CF rates, and univariable model results

	Incident events	Day0 Case fatality	(%)	Day1-27 Case fatality	(%)	Day0 Case fatality				Day1-27 Case fatality			
						
						Rate^†^	p-value	OR	(95% CI)	Rate^†^	p-value	OR	(95% CI)
**All**	372349	178781	(48)	34198	(18)	46				18			
**Gender**													
Men	206841	94107	(45)	15961	(14)	46	< 0.001	REF		17	< 0.001	REF	
Women	165508	84674	(51)	18237	(23)	45		1.25	(1.23,1.26)	19		1.77	(1.74,1.79)
**Age (years)**													
30-44	9572	2519	(26)	165	(2)	26	< 0.001	REF		2	< 0.001	REF	
45-59	54677	16756	(31)	1921	(5)	31		1.23	(1.18,1.28)	5		2.22	(2.06,2.38)
60-74	140890	61980	(44)	11536	(15)	44		2.19	(2.15,2.24)	15		7.14	(6.99,7.30)
75+	167210	97526	(58)	20576	(30)	57		3.91	(3.86,3.95)	29		17.5	(17.4,17.7)
**DEPCAT**													
1 least	16748	8070	(48)	1575	(18)	43	0.211	REF		17	< 0.001	REF	
2	43231	20892	(48)	4169	(19)	44		0.97	(0.92,1.02)	17		1.02	(0.94,1.09)
3	77809	37501	(48)	7446	(18)	45		0.97	(0.92,1.03)	18		1.01	(0.93,1.08)
4	95339	45933	(48)	8853	(18)	46		0.97	(0.91,1.02)	18		0.99	(0.92,1.06)
5	60812	28908	(48)	5513	(17)	46		0.93	(0.88,0.99)	18		0.93	(0.86,1.01)
6	48799	23195	(48)	4257	(17)	47		0.95	(0.89,1.01)	18		0.89	(0.81,0.97)
7 most	29611	14282	(48)	2385	(16)	49		0.94	(0.87,1.01)	17		0.83	(0.74,0.92)
**Year**													
1988-1990	75218	39164	(52)	7690	(21)	51	< 0.001	REF		29	< 0.001	REF	
1991-1993	74672	35830	(48)	7382	(19)	46		0.85	(0.82,0.87)	26		0.86	(0.82,0.89)
1994-1996	66825	31874	(48)	6013	(17)	45		0.83	(0.81,0.86)	23		0.76	(0.72,0.80)
1997-1999	61933	29724	(48)	5231	(16)	45		0.85	(0.82,0.87)	21		0.71	(0.67,0.75)
2000-2002	57002	26150	(46)	4740	(15)	43		0.78	(0.76,0.80)	19		0.66	(0.62,0.70)
2003-2004	36699	16039	(44)	3142	(15)	41		0.71	(0.69,0.74)	18		0.65	(0.61,0.70)

^† ^Rate (per 100) directly age-standardized to the Scottish AMI population

### Geographic variation in case fatality

Table 2 shows the random part estimates from fitting models for each case fatality outcome before and after the inclusion of deprivation. Examination of the intraclass correlation coefficients from each model indicate that the majority (98-99%) of the variation in short-term case fatality is due to differences between individuals, suggesting little geographic variation in short-term survival. The MOR at Level 3 quantifies the differences between HBs, while at Level 2 it quantifies the difference between postcode sectors within the same HB. Focusing on Day0 case fatality, the MOR at each level is 1.16, and the overall MOR, associated with differences between randomly chosen postcode sectors from different HBs, is therefore 1.35 (= 1.16 × 1.16). These geographic inequalities are on a similar scale to, for example, the socioeconomic inequalities experienced by men aged 60+ years (Table 3). When DEPCAT is included in the model, the unexplained heterogeneity (comparing persons from postcode sectors of the same kind; e.g., both areas of low deprivation) does not change much for either of the case fatality outcomes. Therefore, the geographic variation between areas remains even after accounting for small area deprivation.

**Table 2:** Random part (variance) estimates before† and after adjusting for deprivation

	Day0 Case fatality				Day1-27 Case fatality			
	
Level	p-value	Coeff	ICC^‡^	**MOR**^$^	p-value	Coeff	ICC^‡^	**MOR**^$^
*Age, Sex, and Year adjusted*								
HB	0.011	0.024	0.7	1.16	0.014	0.018	0.6	1.14
Postcode	< 0.001	0.024	0.7	1.16	< 0.001	0.008	0.2	1.09
Individual		1	98.6		-	1	99.2	
								
*Age, Sex, Year and DEPCAT adjusted*								
HB	0.011	0.024	0.7	1.16	0.015	0.018	0.5	1.14
Postcode	< 0.001	0.024	0.7	1.16	< 0.001	0.008	0.2	1.09
Individual		1	98.6		-	1	99.3	

^† ^Random part estimates from models including only age, sex, and year of first AMI

^‡ ^Intraclass correlation coefficient (% variance attributable to each level)

^$ ^Median odds ratio

**Table 3:** Age-standardized rates and odds of Day0 case fatality in deprivation categories (ref DEPCAT 1) by age, sex, and year group

	Case fatality rate (per 100) and Odds Ratio (95% CI)											
	
Age (years)	30-59			60+			30-59			60+		
**DEPCAT**	**MEN**						**WOMEN**					

	**1988-1993**											
**All**	32			54			31			51		
**1 (ref)**	28	1		52	1		27	1		51	1	
**2**	30	1.08	(0.90, 1.29)	51	0.96	(0.88, 1.05)	27	1.00	(0.69, 1.46)	50	0.99	(0.91, 1.09)
**3**	30	1.11	(0.94, 1.31)	53	1.06	(0.97, 1.15)	30	1.19	(0.84, 1.67)	51	0.98	(0.90, 1.07)
**4**	34	**1.34**	**(1.14, 1.57)**	55	**1.13**	**(1.04, 1.22)**	30	1.18	(0.84, 1.66)	51	0.98	(0.90, 1.07)
**5**	33	**1.27**	**(1.07, 1.50)**	55	**1.12**	**(1.03, 1.22)**	31	1.27	(0.90, 1.78)	51	0.97	(0.89, 1.06)
**6**	32	**1.21**	**(1.02, 1.43)**	55	**1.17**	**(1.07, 1.28)**	32	1.28	(0.91, 1.81)	51	0.98	(0.90, 1.07)
**7**	38	**1.55**	**(1.30, 1.85)**	58	**1.33**	**(1.21, 1.46)**	34	**1.42**	**(1.00, 2.01)**	53	1.05	(0.96, 1.16)
	**1994-1999**											
**All**	30			50			28			48		
**1 (ref)**	24	1		46	1		20	1		48	1	
**2**	25	1.09	(0.88, 1.35)	49	**1.10**	**(1.00, 1.20)**	25	1.27	(0.80, 2.02)	47	0.95	(0.86, 1.05)
**3**	28	1.19	(0.97, 1.45)	49	**1.10**	**(1.01, 1.20)**	25	1.25	(0.81, 1.93)	47	0.97	(0.89, 1.06)
**4**	30	**1.34**	**(1.10, 1.62)**	51	**1.19**	**(1.09, 1.29)**	28	1.50	(0.98, 2.29)	48	0.98	(0.89, 1.07)
**5**	32	**1.45**	**(1.19, 1.77)**	50	**1.17**	**(1.07, 1.28)**	30	**1.61**	**(1.05, 2.47)**	49	1.02	(0.93, 1.12)
**6**	32	**1.51**	**(1.24, 1.85)**	52	**1.23**	**(1.12, 1.35)**	28	1.49	(0.97, 2.29)	49	1.02	(0.93, 1.12)
**7**	36	**1.76**	**(1.43, 2.16)**	53	**1.35**	**(1.22, 1.49)**	31	**1.74**	**(1.12, 2.71)**	48	0.98	(0.89, 1.09)
	**2000-2004**											
**All**	29			45			28			46		
**1 (ref)**	24	1		41	1		22	1		42	1	
**2**	25	1.06	(0.81, 1.39)	42	1.05	(0.95, 1.18)	28	1.40	(0.79, 2.47)	43	1.00	(0.90, 1.12)
**3**	28	1.22	(0.95, 1.57)	44	**1.13**	**(1.02, 1.25)**	29	1.55	(0.91, 2.66)	45	1.09	(0.98, 1.21)
**4**	30	**1.37**	**(1.07, 1.75)**	45	**1.17**	**(1.06, 1.29)**	26	1.35	(0.80, 2.29)	46	1.08	(0.98, 1.20)
**5**	30	**1.38**	**(1.07, 1.78)**	46	**1.23**	**(1.11, 1.36)**	25	1.35	(0.79, 2.31)	46	1.07	(0.96, 1.19)
**6**	29	**1.34**	**(1.04, 1.73)**	47	**1.29**	**(1.16, 1.44)**	30	1.66	(0.97, 2.85)	47	1.08	(0.97, 1.21)
**7**	34	**1.67**	**(1.28, 2.17)**	50	**1.46**	**(1.30, 1.64)**	32	**1.86**	**(1.08, 3.21)**	47	1.06	(0.94, 1.20)

### Interaction between age, sex, deprivation, and year on Day0 case fatality

There was evidence of a significant four-way interaction (*p*-value < 0.05) between age, sex, year, and DEPCAT for Day0 case fatality. The standardized rates and odds ratios in Table 3 explore the nature of this interaction. To simplify the results, age has been recategorized into two groups (30-59 and 60+ years) and year into three groups (1988-1993, 1994-1999, and 2000-2004). Odds ratios comparing each DEPCAT to DEPCAT1 (most affluent areas) are presented by sex, age, and year group. Significant socioeconomic inequalities existed for men of all ages; these persisted over time and appeared slightly stronger in 30- to 59-year-olds. For example, the odds of case fatality for men aged 30-59 living in the most deprived areas in 2000-2004 were 1.67 (1.28-2.17) times as high as in the least deprived areas. Similar inequalities were apparent in women; however, the odds of case fatality were only significantly greater when comparing the most deprived areas (DEPCAT 7) to the least for women aged 30-59. Again, these inequalities persisted over time. In the younger age group, the odds of case fatality in the most deprived areas were 1.86 (1.08-3.21) times as high as in the least deprived areas.

### Interaction between age, sex, deprivation, and year on Day1-27 case fatality

For Day1-27 case fatality, there was a significant interaction between sex, age, and DEPCAT (*p*-value < 0.05). Table 4 explores the nature of this interaction by, presenting odds ratios comparing each DEPCAT with DEPCAT1 for men and women in each age group. There was little evidence of socioeconomic inequality, with only slightly elevated odds ratios for men aged 60+ living in DEPCATs 3-6; e.g., the odds of case fatality for those men living in DEPCAT 6 were 1.11 (1.00, 1.22) times as high as in DEPCAT 1 (least deprived).

**Table 4:** Age-standardized rates and odds of Day1-27 case fatality in deprivation categories (ref category 1) by age and sex

	Case fatality rate (per 100) and Odds Ratio (95% CI)					
	
Age (years)	30-59			60+		
**DEPCAT**	**MEN**					
**All**	4			20		
**1 (ref)**	4	1		19	1	
**2**	4	0.94	(0.67 1.32)	20	1.07	(0.97 1.18)
**3**	4	1.14	(0.84 1.55)	20	**1.12**	**(1.02 1.23)**
**4**	4	1.09	(0.81 1.48)	20	**1.14**	**(1.04 1.24)**
**5**	5	1.26	(0.93 1.72)	20	**1.11**	**(1.01 1.22)**
**6**	5	1.27	(0.92 1.73)	20	**1.11**	**(1.00 1.22)**
**7**	5	1.37	(0.99 1.91)	19	1.05	(0.94 1.18)
	**WOMEN**					
**All**	7			23		
**1 (ref)**	6	1		22	1	
**2**	7	0.86	(0.50 1.48)	22	1.01	(0.92 1.12)
**3**	7	0.92	(0.55 1.53)	23	1.06	(0.97 1.16)
**4**	7	0.93	(0.56 1.52)	23	1.06	(0.97 1.16)
**5**	7	0.82	(0.50 1.35)	23	1.05	(0.96 1.15)
**6**	6	1.02	(0.61 1.71)	22	1.01	(0.92 1.12)
**7**	7	0.86	(0.51 1.45)	22	0.99	(0.90 1.10)

## Discussion

Unlike other studies of this type, ours examines trends in socioeconomic inequalities in short-term case fatality following a first AMI and the associations with geography, age, and sex. It is the largest population-based study to explore short-term survival from the disease.

### Inequalities in "immediate" case fatality following a first AMI

There has been a steep downward trend in short-term case fatality rates after a first AMI over recent years in Scotland. However, close to one-half of AMI incident events still result in death on the day of the event, implying that a high proportion of these first AMIs are sudden cardiac deaths. There is evidence of socioeconomic inequalities in immediate case fatality [15,29,30]; however, it is unclear whether these gradients differ by age and sex and how these have changed over time. We found socioeconomic inequalities existed in immediate case fatalities in Scotland but varied by age, sex, and time. As hypothesized, the inequalities for men persisted over time and appeared slightly stronger in the younger (30-59 years) age group. Similar inequalities were also evident for women of this age, and these persisted over time when comparing those in the most deprived areas to the least. Although the magnitude of inequalities for this AMI outcome are not as large as generally observed for other outcomes such as coronary heart diseases (CHD) mortality [10] or incidence [2], they are still making an important contribution to the overall picture of CHD inequalities.

We hypothesized that geographical variations in case fatality would be explained by the patterning of socioeconomic deprivation in Scotland. However, after adjusting for area-level deprivation, we found that small but significant geographical variations in case fatality remained. It is logical to think that Day0 case fatalities are mainly sudden cardiac deaths, with limited potential for treatment to be effective. A possible exception is the effect of delays in receiving treatment due to people living in remote areas. Further analysis, not shown here, showed that the significant geographical variation was mainly due to rates being higher in more rural Health Boards. It has been shown elsewhere that short-term case fatality after an AMI is greater in rural areas of Scotland after taking into account deprivation [5], suggesting that a delay in the provision of service is likely to be influential here.

Reducing immediate deaths after AMI incident events requires a reduction in the number of severe first AMIs, which principally requires a focus on primary prevention. Although there is a lack of literature exploring the association between inequalities in AMI risk factor exposure and AMI case fatality, we will discuss which risk factor inequalities have been shown to exist in Scotland and hypothesize any associations with our outcomes. Changes in smoking patterns are likely to be related to changes in immediate case fatality from AMI. Smoking declines over the last 30 years in Scotland have been steeper in men than in women [31], and cigarette smoking prevalence is highest in younger age groups [32]. There are clear socioeconomic inequalities in cigarette smoking, and evidence suggests that gradients are now greater in women than in men in Scotland [32]. The ratio of mean number of cigarettes smoked in the lowest household income quintile compared to the highest was 1.09 in men and 1.51 in women in 2003. A further major risk factor for AMI is obesity, and differences in prevalence are likely to be affecting the severity of first AMI events and hence inequalities in Day0 case fatality. There have been substantial increases in obesity in Scotland over recent years, with higher prevalence in women and the more deprived [32]. Diabetes rates, which are associated with obesity levels, have also been increasing in Scotland [32] and are associated with deprivation, with a suggestion of a stronger gradient in women [32]. There is also evidence that the effect of diabetes on AMI risk is stronger in women than men. Huxley et al [33] found that the association between CHD mortality and diabetes was stronger for women; the pooled relative risk of death was 3.50 for women and 2.06 for men. There is also evidence of a widening of socioeconomic inequalities in high blood pressure in women in the UK [34]. The relationship between cardiovascular risk factors and deprivation is complex and varies across sex and age groups. However, inequalities in risk factors such as those mentioned must be addressed to have a positive effect on Day0 case fatality. Improved primary prevention strategies are vital to reduce the rates overall as these early deaths account for a large proportion (84% in 2003-04) of 28-day case fatality and contribute strongly to Scotland's poor IHD mortality record. Along with reducing incidence rates [2], efforts must be made to reduce the severity of first-time AMIs in Scotland's population.

### Inequalities in 28-day case fatality following a first AMI

There has been a significant drop in 28-day case fatality among those who survive the day of their first AMI event. Such patients will have reached a hospital and received treatment, so reductions in case fatality largely reflect improvements in such treatments. The National Health Service in Scotland provides free health care to all permanent residents, hence we hypothesized that there would be no socioeconomic variations in 28-day case fatality. There was little evidence from our data of such inequalities. As previously mentioned, risk factor exposure is likely to have a stronger effect on immediate case fatality than on 28-day case fatality. A study examining the effect a range of risk factors have on hospitalized case fatality showed a lack of association with some of the common risk factors, such as smoking and high cholesterol and blood pressure [35]. However, high levels of physical activity and moderate drinking were associated with lower case fatality, so variations in these in Scotland may explain the small socioeconomic inequalities in case fatality among those who survived the day of their first AMI.

### Study limitations

One limitation of our study is that we only had an area-based measure of deprivation available and not individual socioeconomic status. The Carstairs deprivation index is a commonly used measure and has been validated against individual socioeconomic status [36]. However, the existing literature lacks information on whether the effect of area-level socioeconomic status in case fatality in Scotland is of relative importance over and above the individual's socioeconomic position. Previous work has shown that population size of the geographic area for which a deprivation index is derived may influence estimation of socioeconomic gradients, whereby estimates of inequalities have been shown to be diluted when the geographical units are large [37]. Our deprivation index is based on areas with a mean population of 5,402, and therefore it is likely our estimates of socioeconomic inequalities are underestimates. It is unfortunate that the routine data used in this study do not permit adjustment for both individual and contextual measures. Further work is needed in the area.

A further limitation is that we do not have individual data on IHD risk factors or comorbidity and therefore can only hypothesize as to why rates are decreasing and inequalities persisting or changing over time. We can also only hypothesize about the contribution reductions and inequalities in AMI case fatality are having on the trends and patterns of AMI mortality in Scotland. It should be noted that our study examines a slightly different hospitalized case fatality outcome than most of the other studies described in this paper. We include patients who have been hospitalized and who have survived the day of their event. We are, therefore, making inferences about a slightly different population group: one that contains fewer of the more severe AMI cases. It should also be noted that we have focused on relative inequalities in case fatality and that inequalities in absolute numbers of incident events resulting in death do differ but are also persisting over time.

## Conclusions

There have been progressive improvements in short-term case fatality from AMI in Scotland. This may reflect improved treatments and a reduction in the incidence of sudden deaths. A high proportion of AMI incident events result in death on the day of the event, mainly sudden cardiac deaths. This highlights the need for primary prevention strategies to reduce risk factor exposure. Socioeconomic inequalities in immediate case fatality were persisting over time in younger men and women, suggesting socioeconomic gradients in risk factor exposure for this age group. In contrast, of those who survive the day of their first AMI, there was little evidence of socioeconomic inequality in 28-day case fatality. This reflects, to some extent, socioeconomic equality in the provision of health care and access to treatment across Scotland.

Inequalities in immediate AMI case fatality suggest that this type of mortality may be highly preventable in Scotland, emphasizing the need for population-wide primary prevention. Reducing case fatality rates in the most disadvantaged populations is key to reducing total AMI mortality in Scotland and would help bring rates to a level comparable with the rest of Western Europe.

## Competing interests

The authors declare that they have no competing interests.

## Authors' contributions

CD is the corresponding author and guarantor of this paper. CD formulated the research question, analysed and interpreted the data and wrote the paper. AL initiated the study and commented on the paper. Both authors read and approved the final manuscript.
